# A Refined Simple First-Order Shear Deformation Theory for Static Bending and Free Vibration Analysis of Advanced Composite Plates

**DOI:** 10.3390/ma12152385

**Published:** 2019-07-26

**Authors:** Hoang Nam Nguyen, Tran Thi Hong, Pham Van Vinh, Nguyen Dinh Quang, Do Van Thom

**Affiliations:** 1 Modeling Evolutionary Algorithms Simulation and Artificial Intelligence, Faculty of Electrical & Electronics Engineering, Ton Duc Thang University, Ho Chi Minh City 700000, Vietnam; nguyenhoangnam@tdtu.edu.vn; 2 Center of Excellence for Automation and Precision Mechanical Engineering, Nguyen Tat Thanh University, Ho Chi Minh City 700000, Vietnam; 3 Faculty of Mechanical Engineering, Le Quy Don Technical University, Hanoi City 100000, Vietnam; 4 Institute of Technology, General Department of Defense Industry, Hanoi City 100000, Vietnam

**Keywords:** Navier solution, simple first-order shear, static bending, free vibration, composite plates

## Abstract

A refined simple first-order shear deformation theory is developed to investigate the static bending and free vibration of advanced composite plates such as functionally graded plates. By introducing the new distribution shape function, the transverse shear strain and shear stress have a parabolic distribution across the thickness of the plates, and they equal zero at the surfaces of the plates. Hence, the new refined theory needs no shear correction factor. The Navier solution is applied to investigate the static bending and free vibration of simply supported advanced composite plates. The proposed theory shows an improvement in calculating the deflections and frequencies of advanced composite plates. The formulation and transformation of the present theory are as simple as the simple first-order shear deformation. The comparisons of deflection, axial stresses, transverse shear stresses, and frequencies of the plates obtained by the proposed theory with published results of different theories are carried out to show the efficiency and accuracy of the new theory. In addition, some discussions on the influence of various parameters such as the power-law index, the slenderness ratio, and the aspect ratio are carried out, which are useful for the design and testing of advanced composite structures.

## 1. Introduction 

Functionally graded materials (FGMs) are a class of advanced composite materials. The mechanical properties of FGMs change continuously over the thickness of structures. In general, FGM is made from a mixture of ceramic and metal. In recent years, they have gained significant attention in many engineering fields such as automotive, civil engineering, aerospace, and nuclear engineering. Hence, due to the exotic properties of FGMs, many researchers have been captivated to investigate the bending behaviors, free vibration, and dynamic and buckling behaviors of FGM beams, plates, and shells. According to the literature, the analysis of FGM plates can be investigated with some different theories such as the classical plate theory (CPT), the first-order shear deformation theory (FSDT), higher-order shear deformation theory (HSDT), the quasi-3D theory and Carrera unified formulation (CUF).

In the CPT, transverse shear deformation is neglected, so only thin plates can be regarded by this theory. Timoshenko et al. [1] used the CPT to analyze plates and shells. Liessa [2] applied the CPT for the free vibration of isotropic thin rectangular plates. Javahenri et al. [3] investigated the buckling behavior of FGM plates under compressive loading. Mohammadi et al. [4] developed analytical solutions based on the Levy procedure to study buckling of FGM plates. In [5], Hu and his co-authors applied the CPT and von Karman assumptions to analyze the vibration and stability of FGM plates, and the influences of some parametric were carried out. In the study of Ghannadpour et al. [6], the buckling of FGM plates under thermal loadings was investigated using the finite strip method based on the CPT. A combination of the CPT and the Rayleigh–Ritz method was used by Chakraverty et al. [7] to analyze the vibration of plates made of FGM. In his work, the plate rested on the Winkler elastic foundation with various boundary conditions. The influence of some parameters of elastic foundation, boundary conditions, and geometric properties were investigated. Kowal-Michalska and his co-authors [8] studied the bending behavior and dynamic buckling of FGM plates using the CPT. In their investigations, the plate was subjected to a combination of thermal and mechanical load. The effects of the neutral surface on the behavior of FGM plates were considered. Damanpack and his colleagues [9] developed a model based on the neutral surface and the CPT to investigate the bending behavior of FGM plates. In their work, the boundary element method was used for numerical computation.

The FSDT developed by Mindlin [10] considered the effects of constant transverse shear deformation, so it can be applied for both thick and thin FGM plates. Raju et al. [11] used the finite element method based on the Mindlin plate theory to study the free vibration of thin and moderately thick plates. Liew et al. [12] applied the Mindlin plate theory to analyze the vibration of thick rectangular plates with different boundary conditions. The bending behaviors of FGM plates were subjected to mechanical load in the thermal environment were investigated by Croce and his partners [13], who used the FSDT. Kim et al. [14] used the FSDT and the Green strain tensor to develop a four-node quasi-conforming shell element which was used to investigate the nonlinear bending behavior of FGM plates and shells. The nonlinear vibration of FGM plates under initial in-plane compressive and bending stresses with a shear deformation effect was studied by Chen [15]. Alijani et al. [16] used the FSDT to study the nonlinear vibration of FGM rectangular plates with movable edges in thermal environments. In his study, the effect of temperature variations and volume coefficients was discussed, and he showed that the deformed FGM plates had stronger hardening behavior in the thermal environment. Fallah and his co-authors [17] employed the FSDT and the extended Kantorovich method to analyze the free vibration of moderately thick FGM plates resting on an elastic foundation. Ganapathi et al. [18] developed a finite element formulation based on the FSDT to study the buckling of FGM skew plates subjected to mechanical loads. Nguyen et al. [19] used the FSDT and the Galerkin method to study the post-buckling of FGM plates with a shear deformation effect; the FGM plates were in a thermal environment and subjected to a mechanical load, and it was found that the behaviour of the plate depended greatly on the temperature. The free vibration of FGM plates and shells were considered by Zhao et al. [20]. In their work, the authors used the element-free kp-Ritz method and the FSDT model. Nguyen et al. [21,22] applied an edge-based smoothed strain smoothing finite element method (ES-FEM) and a node-based smoothed finite element method (NS-FEM) to analyze the static bending, free vibration and buckling behavior of FGM plates. Hosseini-Hashemi and his co-authors [23] developed a Levy solution to investigate the free vibration of FGM rectangular plates. In his extended work [24], a new exact analytical approach was developed based on the Reissner–Mindlin plate theory to analyze the free vibration of FGM rectangular plates. Nguyen et al. [25] developed a new FSDT plate model for the analysis of FGM plates. Singha and his co-authors [26] analyzed FGM plates subjected to transverse load using the FSDT and the finite element method (FEM). The free vibration of isotropic rectangular plates was examined by Manna [27]. In his study, a high-order triangular finite element was developed based on FSDT. The influence of some parameters such as thickness ratios, aspect ratios, and boundary conditions were studied. In the work of Shimpi et al. [28], two refined plate theories (RPT) were developed, and some examples of static bending and free vibration of isotropic plates were carried out. Thai et al. [29,30,31] used a simplified FSDT for FGM plates, and they laminated composite plates and FGM sandwich plates. Senjanović et al. [32] developed a modified Mindlin plate theory for the finite element analysis of thin and thick plates, in which both the bending and shear components of the stiffness matrix were calculated using full integration without shear-looking phenomena. Yu et al. [33] and Yin et al. [34] applied a simplified FSDT for the isogeometric analysis (IGA) of FGM plates. Tan-Van et al. [35] used a simple FSDT-based meshfree method for the static bending and free vibration analysis of FGM plates.

On the other hand, the HSDT developed by Reddy [36] was used by many researchers to study the static bending, free vibration, and buckling of FGM plates. Javaheri et al. [37] applied third-order shear deformation theory (TSDT) and the Navier solution to investigate the thermal buckling of simply supported FGM plates. A nonlinear analysis of FGM plates subjected to transverse loads in the thermal environment was investigated by Shen et al. [38,39] using the TSDT. Yang et al. [40] studied the buckling, free vibration and dynamic stability of laminated FGM plates using the HSDT. Yang and his co-authors [40] studied the free vibration, buckling behavior, and dynamic stability of laminated FGM plates using the HSDT. Yang et al. [41] and Huang et al. [42] employed the TSDT to investigate the free vibration, transient response, and nonlinear vibration of initially stressed FGM plates, and they found that the material properties of the plate depended on the temperature. Bodaghi and his partners [43] developed an analytical solution for the buckling of thick FGM rectangular plates under in-plane loadings based on the HSDT. Ferreira et al. [44,45] studied static bending of FGM plates using the TSDT and a robust meshless collocation method. Thai and his colleagues [46] used a neutral surface based-TSDT to analyze the buckling of FGM plates. Kim [47] employed the TSDT and the Rayleigh–Ritz procedure to investigate the vibration of FGM plates, in which the material properties depended on the temperature. Hosseini-Hashemi et al. [48] used the TSDT to derive a new exact solution to analyze the free vibration of FGM rectangular plates. Baferani et al. [49] developed an accurate solution based on FSDT and the Levy solution to analyze the free vibration of FGM plates. The work focused on the effect of the elastic foundation on the behavior of FGM plates. Tran et al. [50] analyzed FGM plates using the HSDT and IGA. Do et al. [51] investigated the influences of material combination and thermal environment in the mechanical behavior of FGM sandwich plates. Zenkour [52] developed a generalized shear deformation theory for the bending analysis of FGM plates. Senthilnathan et al. [53] and Murty [54] employed a simplified TSDT for the analysis of laminated composite plates. Shimpi [55] used the RPT and its variants for analysis of isotropic and orthotropic plates. Thai et al. [56,57,58] developed various HSDTs for the bending, buckling and vibration of FGM plates. Mechab et al. [59] proposed a four-variable refined plate theory based on an HSDT for the static and dynamic analysis of FGM plates. Meiche et al. [60] developed a new four-unknown HSDT using a hyperbolic shear function for the buckling and vibration of FGM sandwich plates. Nguyen-Xuan and his co-authors [61] developed a refined plate theory based on the HSDT for the isogeometric analysis of FGM plates. Zhang et al. [62] used the TSDT to investigate the nonlinear dynamics and chaotic vibration of a simply supported orthotropic FGM rectangular plate in the thermal environment subjected to parametric and external excitations. Hao and his co-authors [63] studied the nonlinear oscillation of a cantilever FGM rectangular plate subjected to the transversal excitation in the thermal environment using the TSDT and an asymptotic perturbation method. Wang et al. [64] applied sinusoidal shear deformation theory (SSDT) to focus on performing a free vibration analysis of a FGM porous cylindrical shell with different sets of boundary conditions. Wang and his colleagues [65] developed a new HSDT to analyze the forced vibration of an FG graphene nanoplatelet reinforced composite beam under two successive moving masses.

Recently, a quasi-3D theory has been developed to study medium, thick, and very thick FGM plates. This theory accounts for higher-order variations of both in-plane and transverse displacement across the thickness and, consequently, takes the effects of both shear and normal deformations. Pandya and Kant [66] developed a finite element formulation based on a seven-unknown HSDT for the flexure of sandwich plates. Touratier [67] studied isotropic and laminated composite plates, an investigation in which the author developed a SSDT with five unknowns. Soldatos [68] analyzed a homogeneous monoclinic plate using the HSDT with hyperbolic shear function. Werner [69] developed a three-dimensional solution for rectangular plate bending. Batra and Vidoli [70] used a three-dimensional variational principle to derive an HSDT for the analysis of piezoelectric plates. Qian et al. [71,72] applied the HSDT and the normal deformable plate theory and meshless local Petrov–Galerkin (MLPG) method for the static bending, free vibration, and dynamic response of FGM plates. Gilhooley et al. [73] also used the HSDT and the normal deformable plate theory and MLPG with radial basis functions for the analysis of thick FGM plates. Talha and his co-authors [74] used the HSDT to study the bending behavior and free vibration of FGM plates—the effect of some geometric parameters and the power-law index were carried out. Nguyen et al. [75] applied the HSDT and IGA for the analysis of composite sandwich plates. Akavci [76,77] developed two new hyperbolic HSDTs for the analysis of laminated composite and FGM plates. Karama and his partners [78] employed the HSDT for analysis of laminated composite beams. In this study, the composite beam was modelled by the multi-layered model based on the HSDT. Matsunaga [79] analyzed the free vibration and stability of FGM plates. In his work, the FGM plates were modelled using a 2-D HSDT. Aydogdu [80] developed a new HSDT to analyze laminated composite plates. Mantari and his co-authors [81,82,83,84,85,86,87,88,89,90] developed various quasi-3D plate theories for the static bending, free vibration, and buckling of laminated composite plates, FGM plates, and sandwich FGM plates. Nguyen et al. [91] developed a new inverse trigonometric shear deformation theory for isotropic and FGM sandwich plates analysis. Thai et al. [92,93] applied IGA with the inverse trigonometric shear deformation theory and generalized shear deformation theory to investigate laminated composite and FGM sandwich plates. Zenkour [94] used the sinusoidal function to develop 3-D elasticity solutions to study bending behavior and free vibration of exponentially graded thick rectangular plates. Bui et al. [95] applied the TSDT and the FEM for the mechanical behaviors of heated FGM plates in a high-temperature environment. Do et al. [96] analyzed bi-directional FGM plates using the FEM and the TSDT. Mantari et al. [97,98] developed various quasi-3D theories which consisted of four unknowns to study FGM plates. Thai et al. [99] employed a sinusoidal function to develop a simple quasi-3D theory with only five unknowns to analyze FGM plates. Zenkour [100,101,102,103] developed many different quasi-3D theories which contained only four unknowns to study the bending behavior and vibration behavior of FGM plates and FGM sandwich plates. Neves and his co-authors [104] developed a new quasi-3D theory using a hyperbolic function to analyze FGM plates. Neves et al. [105] applied a quasi-3D HSDT and a meshless technique for the static bending, free vibration and buckling of sandwich FGM plates. In [106], Neves and his co-authors developed a quasi-3D SSDT to analyze FGM plates. Cerrera et al. [107] investigated the influences of thickness stretching in FGM plates and shells.

Furthermore, Carrera et al. [108,109] proposed the unified formulation (CUF) for multilayered composite structures. Brischetto et al. [110,111] studied the bending behavior of FGM plates and shells using CUF. Cinefra et al. [112] and Ferreira et al. [113] investigated the bending behavior and vibration behavior of laminated composite shells. In their works, the SSDT was developed using CUF. The bending behavior of FGM plates and shells was investigated by Cinefra and his co-authors [114]. In his work, the combination of the CUF and the mixed interpolation of tensorial components (MITC) was used to develop a nine-node shell element.

By decomposing the transverse displacements into two parts, the bending part and shear part, the simplified FSDT has less unknowns than the FSDT, the HSDT, the SSDT and the quasi-3D theory, so its computational expenses are reduced. Thus, the development of a simplified FSDT is still necessary. This paper developed a refined simple FSDT for the analysis of advanced composite plates, such as FGM plates. By introducing the distribution shape function to the shear strain, the proposed theory not only shows an improvement on expecting deflections but also accounts for a parabolic transverse shear strain distribution through the thickness of the plates. The Navier solution was applied to investigate the static bending and free vibration of simply supported plates. Several numerical examples are presented to illustrate the accuracy of the new refined plate theory.

## 2. Material Properties of Advanced Composite Plates

**Figure 1:**
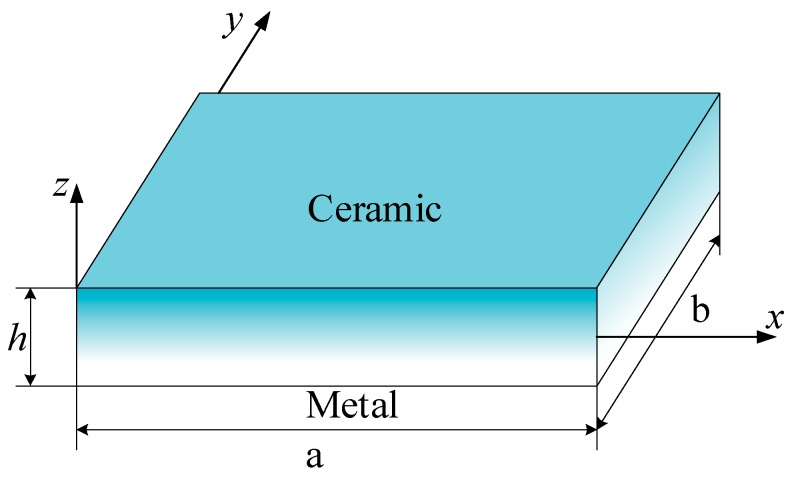
Functionally graded materials (FGM) plate model.

Advanced composite materials such as functionally graded materials can be produced by continuously varying the constituents of multi-phase materials in a predetermined profile. An FGM can be defined by the variation in the volume fractions. In this paper, FGM plates with the power-law function (P-FGM) and exponential function (E-FGM) were considered (Figure 1). 

For the case of P-FGM plates, the materials properties of P-FGM depend on the volume fraction, which can be obtained as a power-law function as the following formula.

(1) $$V_{c}={\left(\frac{1}{2}+\frac{z}{h}\right)}^{p}$$

where *p* is the material parameter and h is the thickness of the plate. The material properties of a P-FGM can be determined as

(2) $$P(z)=P_{m}+(P_{c}-P_{m})V_{c}$$

where $P_{c},\;P_{m}$ are the Young’s modulus or density of the ceramic and metal, respectively. 

For the case of E-FGM plates, the material properties of E-FGM are defined as

(3) $$P(z)=P_{0}e^{p(z+h/2)}$$

where $P_{0}$ is the Young’s modulus or density of the bottom surface of the FGM plate and p is the material parameter. 

## 3. Formulation of Refined First-Order Shear Deformation Theory

### 3.1. Kinematics

Corresponding to the simple FSDT, the transverse displacement w is separated into two parts—the bending constituent $w_{b}$ and the shear constituent $w_{s}$. The displacement fields of the plate can be expressed as

(4) $$\begin{array}{l} u=u-z\frac{\partial w_{b}}{\partial x}\\ v=v-z\frac{\partial w_{b}}{\partial y}\\ w=w_{b}+w_{s} \end{array}$$

The strains related to the displacement fields are

(5) $$\begin{array}{l} \varepsilon_{x}=\frac{\partial u}{\partial x}-z\frac{\partial^{2}w_{b}}{\partial x^{2}}\\ \varepsilon_{y}=\frac{\partial v}{\partial y}-z\frac{\partial^{2}w_{b}}{\partial y^{2}}\\ \gamma_{xy}=\frac{\partial u}{\partial y}+\frac{\partial v}{\partial x}-2z\frac{\partial^{2}w_{b}}{\partial x\partial y}\\ \gamma_{xz}=\frac{\partial w_{s}}{\partial x}\\ \gamma_{yz}=\frac{\partial w_{s}}{\partial y} \end{array}$$

Certainly, the simple FSDT theory was based on the statement of linear shear strain distribution across thickness, so a constant shear correction coefficient was needed to overcome the shear-locking phenomenon. Nevertheless, the shear stress was distributed parabolically across the thickness and disappeared on the top and bottom surfaces of the plate. In this paper, an assumption of shear distributed function is presented to improve the simple FSDT. Therefore, the shear strains vector becomes

(6) $$\left\{\begin{array}{l} \gamma_{xz}^{c}\\ \gamma_{yz}^{c} \end{array}\right\}=f(z)\left\{\begin{array}{l} \gamma_{xz}\\ \gamma_{yz} \end{array}\right\}$$

where f(z) is the assuming shear distributed function, which defines the distribution of the transverse shear strains across the thickness of the plate. The shear distributed function was chosen so it satisfies the following conditions: The shear strain is distributed parabolically over the thickness and equal to zero on the top and bottom surfaces of the plate; the integration through the thickness of the plate approximating with the constant shear correction factor of the FSDT (5/6). Inspired by the study of Zenkour [52], the shear distributed function can be chosen as

(7) $$f(z)=\frac{5}{4}\cos\left(\frac{\pi z}{h}\right)$$

The constitutive equations for the plate can be expressed as

(8) $$\left\{\begin{array}{l} \sigma_{x}\\ \sigma_{y}\\ \tau_{xy}\\ \tau_{xz}\\ \tau_{yz} \end{array}\right\}=\frac{E(z)}{1-\nu^{2}}\left[\begin{array}{lllll} 1 & \nu & 0 & 0 & 0\\ \nu & 1 & 0 & 0 & 0\\ 0 & 0 & \frac{1-\nu }{2} & 0 & 0\\ 0 & 0 & 0 & \frac{1-\nu }{2} & 0\\ 0 & 0 & 0 & 0 & \frac{1-\nu }{2} \end{array}\right]\left\{\begin{array}{l} \varepsilon_{x}\\ \varepsilon_{y}\\ \gamma_{xy}\\ \gamma_{xz}\\ \gamma_{yz} \end{array}\right\}$$

### 3.2. Equations of Motion

The equations of motion can be quantified using the Hamilton’s principle, that is

(9) $$0=\int_{0}^{T}\left(\delta U+\delta V-\delta K\right)dt$$

where δU is the variation of strain energy, δV is the variation of work done by external forces, and δK is the variation of kinetic energy. The expression of δU is

(10) $$\delta U=\int_{A}^{}\int_{-h/2}^{h/2}\left(\sigma_{x}\delta \varepsilon_{x}+\sigma_{y}\delta \varepsilon_{y}+\tau_{xy}\delta \gamma_{xy}+\tau_{xz}^{c}\delta \gamma_{xz}^{c}+\tau_{yz}^{c}\delta \gamma_{yz}^{c}\right)dzdA$$

(11) $$\begin{array}{l} \delta U=\int_{A}^{}[N_{x}\frac{\partial \delta u}{\partial x}-M_{x}\frac{\partial^{2}\delta w_{b}}{\partial x^{2}}+N_{y}\frac{\partial \delta v}{\partial y}-M_{y}\frac{\partial^{2}\delta w_{b}}{\partial y^{2}}+\\ +N_{xy}\left(\frac{\partial \delta u}{\partial y}+\frac{\partial \delta v}{\partial x}\right)-2M_{xy}\frac{\partial^{2}\delta w_{b}}{\partial x\partial y}+Q_{x}^{c}\frac{\partial \delta w_{s}}{\partial x}+Q_{y}^{c}\frac{\partial \delta w_{s}}{\partial y}]dA \end{array}$$

where N, M, and $Q^{c}$ are the stress resultants which are defined by

(12) $$\left(N_{x},N_{y},N_{xy}\right)=\int_{-h/2}^{h/2}\left(\sigma_{x},\;\sigma_{y},\;\sigma_{xy}\right)dz$$

(13) $$\left(M_{x},M_{y},M_{xy}\right)=\int_{-h/2}^{h/2}\left(\sigma_{x},\;\sigma_{y},\;\sigma_{xy}\right)zdz$$

(14) $$\left(Q_{x}^{c},Q_{y}^{c}\right)=\int_{-h/2}^{h/2}\left(\tau_{xz}^{c},\;\tau_{yz}^{c}\right)f(z)dz$$

The expression of δV is

(15) $$\delta V=-\int_{A}^{}q\delta \left(w_{b}+w_{s}\right)dA$$

The expression of the variation of kinetic energy δK is

(16) $$\delta K=\int_{V}^{}\left(\dot{u}\delta \dot{u}+\dot{v}\delta \dot{v}+\dot{w}\delta \dot{w}\right)\rho (z)dV$$

After integrating Equation (16) over the thickness direction, Equation (16) becomes

(17) $$\begin{array}{l} \delta K=\int_{A}^{}\{I_{0}\left[\dot{u}\delta \dot{u}+\dot{v}\delta \dot{v}+\left({\dot{w}}_{b}+{\dot{w}}_{s}\right)\delta \left({\dot{w}}_{b}+{\dot{w}}_{s}\right)\right]\\ -I_{1}\left(\dot{u}\frac{\partial \delta {\dot{w}}_{b}}{\partial x}+\frac{\partial {\dot{w}}_{b}}{\partial x}\delta \dot{u}+\dot{v}\frac{\partial \delta {\dot{w}}_{b}}{\partial y}+\frac{\partial {\dot{w}}_{b}}{\partial y}\delta \dot{v}\right)\\ +I_{2}\left(\frac{\partial {\dot{w}}_{b}}{\partial x}\frac{\partial \delta {\dot{w}}_{b}}{\partial x}+\frac{\partial {\dot{w}}_{b}}{\partial y}\frac{\partial \delta {\dot{w}}_{b}}{\partial y}\right)\}dA \end{array}$$

where

(18) $$\left(I_{0},I_{1},I_{2}\right)=\int_{-h/2}^{h/2}\left(1,z,z^{2}\right)\rho (z)dz$$

Substituting Equations (11), (15) and (17) into Equation (9) and integrating by parts, the equations of motions are obtained as

(19) $$\delta u:\frac{\partial N_{x}}{\partial x}+\frac{\partial N_{xy}}{\partial y}=I_{0}\ddot{u}-I_{1}\frac{\partial {\ddot{w}}_{b}}{\partial x}$$

(20) $$\delta v:\frac{\partial N_{y}}{\partial y}+\frac{\partial N_{xy}}{\partial x}=I_{0}\ddot{v}-I_{1}\frac{\partial {\ddot{w}}_{b}}{\partial y}$$

(21) $$\delta w_{b}:\frac{\partial^{2}M_{x}}{\partial x^{2}}+\frac{\partial^{2}M_{y}}{\partial y^{2}}+2\frac{\partial M_{xy}}{\partial x\partial y}+q=I_{0}\left({\ddot{w}}_{b}+{\ddot{w}}_{s}\right)+I_{1}\left(\frac{\partial \ddot{u}}{\partial x}+\frac{\partial \ddot{v}}{\partial y}\right)-I_{2}\nabla^{2}{\ddot{w}}_{b}$$

(22) $$\delta w_{s}:\frac{\partial Q_{x}^{c}}{\partial x}+\frac{\partial Q_{y}^{c}}{\partial y}+q=I_{0}\left({\ddot{w}}_{b}+{\ddot{w}}_{s}\right)$$

where $\nabla^{2}=\frac{\partial^{2}}{\partial x^{2}}+\frac{\partial^{2}}{\partial y^{2}}$.

## 4. Analytical Solutions

In this study, a simply supported rectangular plate was considered. The length of the plate was a, the width of the plate was b, and the height of the plate was h. The plate was subjected to a distributed transverse load q. Employing the Navier solution, the solutions of the plate were assumed as

(23) $$\left\{\begin{array}{c} u(x,y,t)\\ v(x,y,t)\\ w_{b}(x,y,t)\\ w_{s}(x,y,t) \end{array}\right\}=\sum_{m=1}^{\infty }\sum_{n=1}^{\infty }\left\{\begin{array}{c} U_{mn}e^{i\omega t}\cos\alpha_{m}x\sin\beta_{n}y\\ V_{mn}e^{i\omega t}\sin\alpha_{m}x\cos\beta_{n}y\\ W_{bmn}e^{i\omega t}\sin\alpha_{m}x\sin\beta_{n}y\\ W_{smn}e^{i\omega t}\sin\alpha_{m}x\sin\beta_{n}y \end{array}\right\}$$

where $i^{2}=-1,$ $\alpha_{m}=m\pi /a,$ $\beta_{n}=n\pi /b,$ $\left(U_{mn},\;V_{mn},\;W_{bmn},\;W_{smn}\right)$ are quantities to be determined, m and n are mode numbers, and ω is the frequency of free vibration. The transverse distributed load q was also expanded in the following form

(24) $$q(x,y)=\sum_{m=1}^{\infty }\sum_{n=1}^{\infty }Q_{mn}\sin\alpha_{m}x\sin\beta_{n}y$$

For the case of a sinusoidal distributed load, we have

(25) $$Q_{11}=q_{0},\;m=n=1$$

For the case of uniformly distributed load, the coefficients $Q_{mn}$ are defined as follows

(26) $$Q_{mn}=\frac{16q_{0}}{mn\pi^{2}}$$

By substituting Equations (23) and (24) into the equations of motion, Equations (19)–(22), analytical solutions can be obtained from the following equation.

(27) $$\left(K-\omega^{2}M\right)\Delta =f$$

where K and M are, respectively, the stiffness matrix and the mass matrix; f is the force vector; Δ is the vector of unknown coefficients, and ω is the frequency of free vibration. The elements of the K, M, f, and Δ are as follows

(28) $$\begin{array}{l} k_{11}=A_{11}\alpha^{2}+A_{33}\beta^{2},\;k_{12}=(A_{12}+A_{33})\alpha \beta ,\\ k_{13}=-B_{11}\alpha^{3}-(B_{12}+2B_{33})\alpha \beta^{2},\;k_{22}=A_{22}\beta^{2}+A_{33}\alpha^{2},\\ k_{23}=-B_{22}\beta^{2}-(B_{12}+2B_{33})\alpha^{2}\beta ,\;k_{33}=D_{11}\alpha^{4}+(2D_{12}+4D_{33}),\alpha^{2}\beta^{2}+D_{22}\beta^{4}\\ k_{44}=As_{11}\alpha^{2}+As_{22}\beta^{2},\;k_{14}=k_{24}=k_{34}=0, \end{array}$$

(29) $$\begin{array}{l} m_{11}=I_{0},\;m_{13}=-\alpha I_{1},\;m_{22}=I_{0},\;m_{23}=-\beta I_{1},\;m_{33}=I_{0}+I_{2}(\alpha^{2}+\beta^{2}),\\ m_{34}=I_{0},\;m_{44}=I_{0},\;m_{12}=m_{14}=m_{24}=0, \end{array}$$

(30) $$f_{1}=f_{2}=0,\;f_{3}=f_{4}=Q_{mn},$$

(31) $$\Delta ={\left\{U_{mn},\;V_{mn},\;W_{bmn},\;W_{smn}\right\}}^{T}.$$

For bending analysis, the closed-form solution could be obtained by setting the natural frequency ω equal to zero. For free vibration analysis, the closed-form solution was obtained by setting the transverse load q equal to zero.

## 5. Numerical Results and Discussion

In this section, some numerical illustrations are carried out and discussed to prove the efficiency and accuracy of the proposed theory in the static bending and free vibration responses of simply supported isotropic homogeneous and FGM plates. The non-dimensional entities were used as the following formulas

(32) $$\bar{w}=\frac{10E_{c}h^{3}}{q_{0}a^{4}}w\left(\frac{a}{2},\frac{b}{2}\right),{\bar{\sigma }}_{x}(z)=\frac{h}{q_{0}a}\sigma_{x}\left(\frac{a}{2},\frac{b}{2},z\right),{\bar{\sigma }}_{y}(z)=\frac{h}{q_{0}a}\sigma_{y}\left(\frac{a}{2},\frac{b}{2},z\right),\;{\bar{\sigma }}_{xy}(z)=\frac{h}{q_{0}a}\sigma_{xy}\left(0,0,z\right),{\bar{\sigma }}_{xz}(z)=\frac{h}{q_{0}a}\sigma_{xz}\left(0,\frac{b}{2},z\right),{\bar{\sigma }}_{yz}(z)=\frac{h}{q_{0}a}\sigma_{xz}\left(\frac{a}{2},0,z\right),\;\hat{\omega }=\omega h\sqrt{\frac{\rho_{c}}{E_{c}}},\tilde{\omega }=\omega {\left(\frac{a}{h}\right)}^{2}\sqrt{\frac{\rho_{c}}{E_{c}}},\bar{\omega }=\omega \frac{a^{2}}{h}\sqrt{\frac{\rho_{c}}{E_{c}}},\omega^{*}=\omega \frac{a^{2}}{h\pi^{2}}\sqrt{\frac{12\rho }{E}}.$$

### 5.1. Static Bending Analysis

**Example** **1.** Firstly, the results obtained using the present theory were compared with those of the classical plate theory [1] given by Timoshenko, the Navier-type three-dimensionally (3-D) exact solution given by Werner [69], and the generalized shear deformation theory by Zenkour [52] in Table 1 and Table 2. The geometric and material properties of plate were a=1, b=1, E=1, $q_{0}=1,$ ν=0.3 with three cases of the thickness of plate h=0.01, h=0.03, and h=0.1. The comparison exhibited the fact that that the present results were in good agreement with other published results. According to Table 2, the axial stress equaled zero at the mid-plane for the case of the isotropic plate. Therefore, the neutral surface was identical to mid-plane for the isotropic plate.

**Example** **2.** Next, a functionally graded square plate made of aluminum (Al) and alumina (Al_2_O_3_) subjected to a uniform or sinusoidal distributed load was considered. The Young’s modulus for (Al) was 70 GPa and 380 GPa for Al_2_O_3_, while Poisson’s ratios were constant for both, equaling 0.3. Young’s modulus was calculated using the power-law distribution. The solutions obtained using the present theory were compared with the solutions of Zenkour [52] using the generalized shear deformation theory with different values of the power-law index p and the constant value of ratio a/h=10. The comparisons are given in Table 3 and Table 4. According to these tables, the solutions of the proposed plate theory were very close to the results of Zenkour [52].

**Figure 2:**
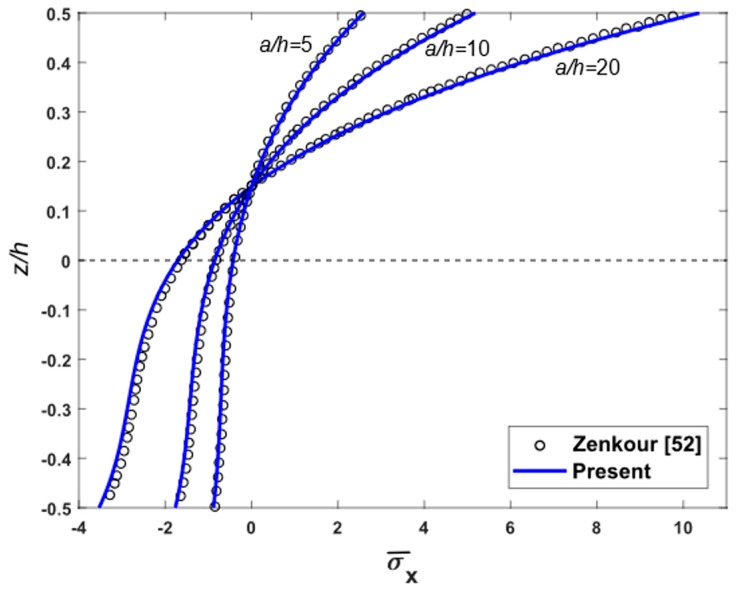
Distribution of non-dimensional axial stresses ${\bar{\sigma }}_{x}$ across the depth of isotropic Al/Al_2_O_3_ plates subjected to uniform load for some cases of side-to-thickness ratio.

**Figure 3:**
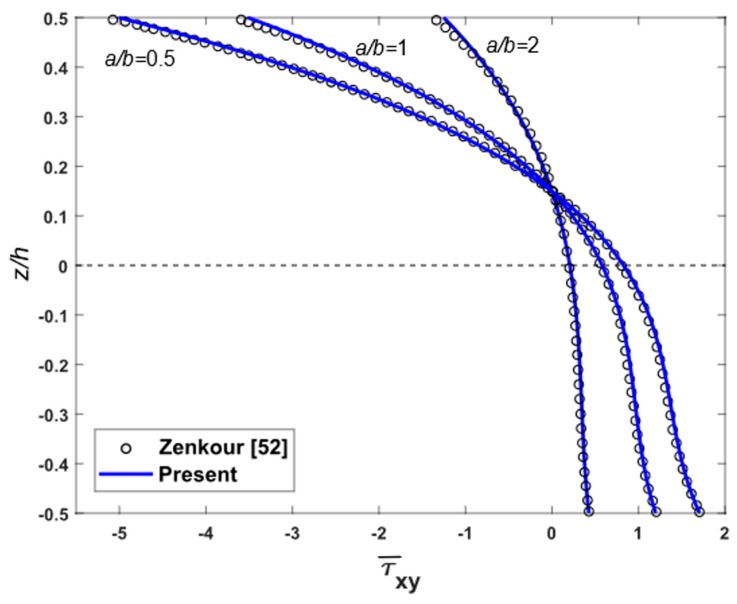
Distribution of non-dimensional shear stresses ${\bar{\tau }}_{xy}$ across the thickness of isotropic Al/Al_2_O_3_ plates subjected to uniform loads for some cases of aspect ratio.

**Figure 4:**
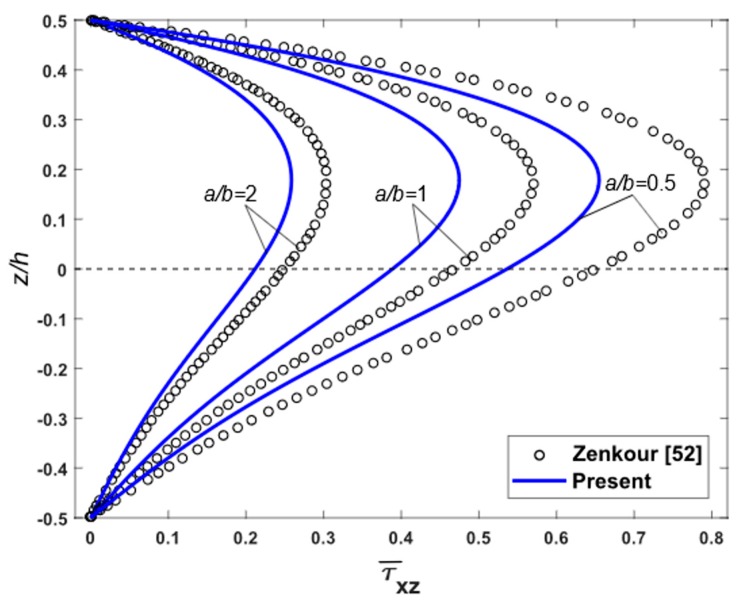
Distribution of non-dimensional shear stresses ${\bar{\tau }}_{xz}$ across the thickness of isotropic Al/Al_2_O_3_ plates subjected to uniform loads for various values of aspect ratio.

**Figure 5:**
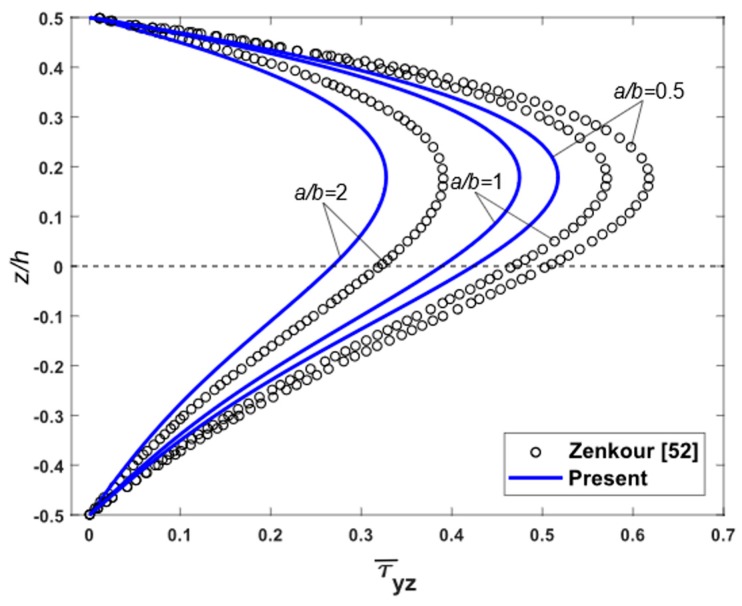
Distribution of non-dimensional shear stresses ${\bar{\tau }}_{yz}$ across the thickness of isotropic Al/Al_2_O_3_ plates subjected to uniform loads for various values of aspect ratio.

**Figure 6:**
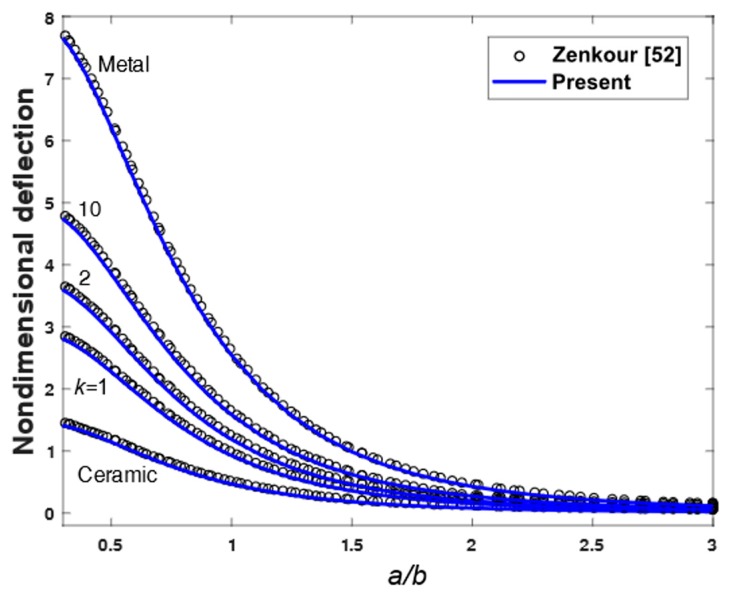
Non-dimensional center deflection as a function of the aspect ratio (*a/b*) of an FGM plate.

**Figure 7:**
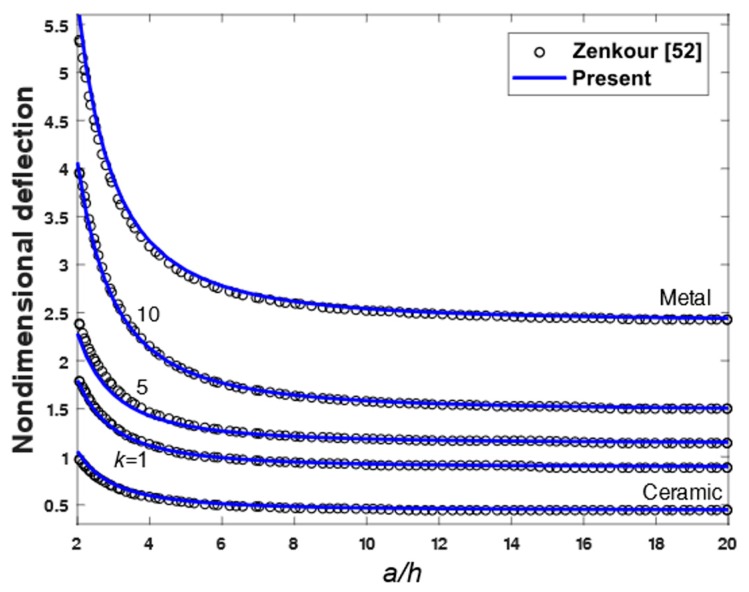
Non-dimensional center deflection as a function of the side-to-thickness ratio (*a*/*h*) of an FGM plate.

To demonstrate the accuracy of the present theory for wide range of aspects and side-to-thickness ratios a/h, through the thickness distributions of the in-plane longitudinal and normal stresses ${\bar{\sigma }}_{x}$ and ${\bar{\sigma }}_{y}$, the longitudinal tangential stress ${\bar{\tau }}_{xy}$, the shear stresses ${\bar{\tau }}_{xz}$ and ${\bar{\tau }}_{yz}$ in the FGM plate under uniform load are explained in Figure 2, Figure 3, Figure 4, Figure 5, Figure 6 and Figure 7, which show, respectively, the influence of the aspect ratio and the side-to-thickness ratio on the center deflection of the plates. The obtained results were compared with those reported by Zenkour [52]. The comparison shows that the results of the present theory and Zenkour are almost identical, except for the case of the transverse shear stresses ${\bar{\tau }}_{xz}$ and ${\bar{\tau }}_{yz}$, where a small difference between the results can be seen. However, it should be noticed that the results of Zenkour were obtained using the generalized shear deformation theory, while the present results were obtained using the proposed refined simple FSDT. According to Figure 2, the axial stress did not equal to zero at the mid-plane of the FGM plates, so the neutral surface moved toward the ceramic surface of the FGM plates. From Figure 4 and Figure 5, the shear stresses were asymmetric through the thickness of the FGM plates. In addition, Figure 6 and Figure 7 show that the deflection of the plate decreased when the aspect ratio (*a/b*) and side-to-thickness ratio (*a/h*) increased.

**Example** **3.** In the next example, a square plate made of Al/Al_2_O_3_ was considered. The plate was subjected to a sinusoidal distributed load. Young’s modulus for Al was 70 GPa and 380 GPa for Al_2_O_3_, while Poisson’s ratios were constant for both and equal to 0.3. Young’s modulus was expressed by Equation (2). Three different values of the power-law index p=1, p=4 and p=10 were used in this example. The results obtained using the present theory were compared with the solutions given by Neves et al. [104,105,106], Carrera et al. [107,108], and Thai et al. [29], in which Neves and Carrera used different quasi-3D theories and Thai used a simple FSDT. In addition, it should be observed that the effect of thickness stretching is accounted in quasi-3D theories, while it is ignored in the simple FSDT of Thai and their proposed theory. According to Table 5, it can be noticed that results obtained of the present theory are in good agreement with published results for both thin and thick FGM plates.

**Example** **4.** Continuously, an exponential FGM plate with thickness ratio a/h=2 and a/h=4 were investigated. The Poisson’s ratios were constant and equal to 0.3. Young’s modulus was evaluated using the exponential distribution. The results of the present theory were compared with those of the 3D elasticity solution [94], quasi-3D theories [88,94], the HSDT [85], and the simple HSDT [56]. From Table 6, the present results are in excellent agreement with literature results for medium thick plates. For the very thick FGM plates (a/h=2), the deflections obtained of the proposed theory were slightly larger than those of 3D results and quasi-3D results, because the thickness stretching effect was neglected in the present theory.

### 5.2. Free Vibration Analysis

**Example** **5.** The next verification was performed for the free vibration of an isotropic homogeneous rectangular plate with a simply supported boundary condition. The length-to-height ratios of the plates were a/h=1000 and 5. The first six non-dimensional frequencies $\omega^{*}$ of the present theory were compared with the available published results of Manna [27], Leissa [2], Liew et al. [12] and Raju [11], in which, Manna [27] used a family of higher-order triangular element, Leissa [2] used an analytical solution, Liew et al. [12] used the pb-2 Rayleigh–Ritz method, and Raju [11] used a nine-node Lagrangian quadrilateral isoparametric plate element. The comparision was shown in Table 7. According to Table 7, it can be concluded that the present solutions are in good agreement with published solutions.

**Example** **6.** The next example was carried out for an isotropic Al/Al_2_O_3_ square plate. The Young’s modulus and density of aluminum were *E_m_* = 70 GPa and $\rho_{m}$ = 2702 kg/m^3^, respectively, and those of alumina were *E_c_* = 380 GPa and $\rho_{c}$ = 3800 kg/m^3^, respectively. The Poisson’s ratio of the plate was assumed to be constant through the thickness, and it equaled 0.3. In this example, Young’s modulus and density were obtained using Equation (2). The length-to-thickness ratio a/h varied from 2 to 10, and the power-law index varied from 0 to 10. The first two non-dimensional frequencies $\hat{\omega }$ for different values of length-to-thickness ratio a/h and the power-law index p using the present theory and those of other theories are given in Table 8. From this table, it can be found that the present theory has an excellent accuracy to determine the frequency for FGM plates. It was also observed that the non-dimensional frequencies of FGM plates decreased as the value of the power-law index increased.

**Figure 8:**
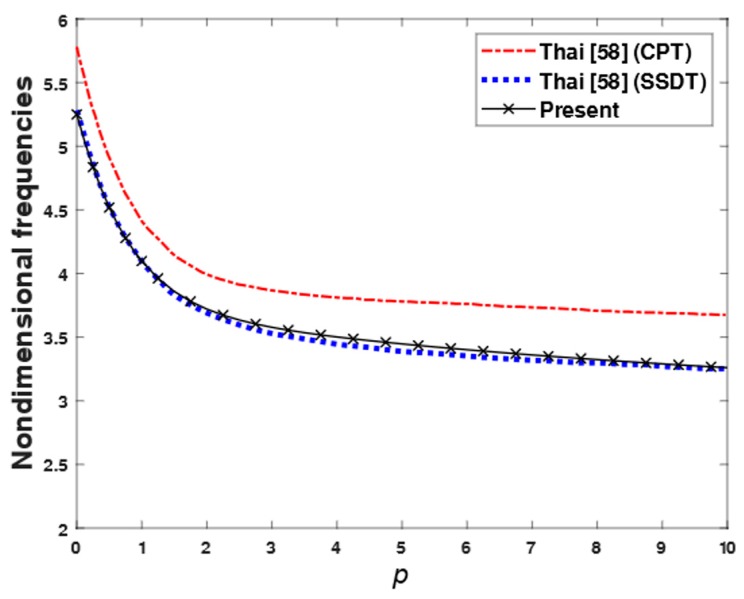
The variation of first frequencies on the power-law index.

**Figure 9:**
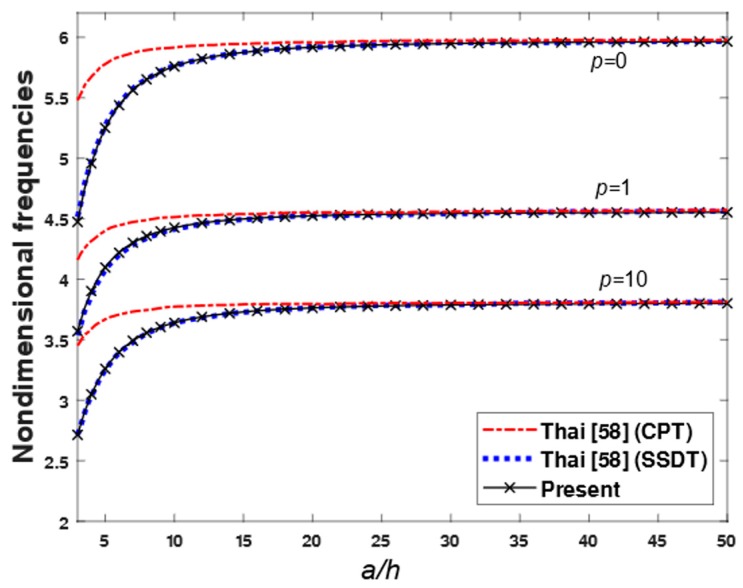
The effect of length-to-thickness on the nondimensional first frequencies.

**Example** **7.** The first four non-dimensional frequencies $\bar{\omega }$ of an FGM rectangular plate with length-to-thickness ratio varied from 5 to 20 and the power-law index varied from 0 to 10 are compared in Table 9. The plate was made from aluminum (as metal) and alumina (as ceramic). The material properties of aluminum were *E_m_* = 70 GPa and $\rho_{m}$ = 2702 kg/m^3^, and those of alumina were *E_c_* = 380 GPa and $\rho_{c}$ = 380 kg/m^3^. The Poisson’s ratio of the plate was assumed to be constant through the thickness, and it equaled to 0.3. Equation (2) was used to evaluate the Young’s modulus and density of the plate. The first four non-dimensional frequencies $\bar{\omega }$ obtained by using the present theory were compared with those given by Hosseini-Hashemi et al. [24] based on the FSDT, Reddy [36] based on the TSDT, and Thai et al. [58] based on the SSDT. In addition, the variations of the non-dimensional fundamental frequency of FGM square plate with respect to the power-law index p and length-to-thickness ratio a/h are compared in Figure 8 and Figure 9, respectively. According to Table 9 and Figure 8 and Figure 9, the non-dimensional frequencies achieved by the proposed theory are in excellent agreement with those obtained by the FSDT, TSDT and SSDT. From Table 9 and Figure 8, the first frequencies of the FGM plate decreased when the power-law index increased. When the length-to-thickness ratio increased, the first frequencies of the FGM plate increased, as shown in Figure 9.

**Example** **8.** This example aimed to verify the obtained results of thin and thick plates. A fully simply supported Al/Al_2_O_3_ square thick plate with different length-to-thickness ratios a/h was analyzed. The material properties of aluminum were *E_m_* = 70 GPa and $\rho_{m}$ = 2707 kg/m^3^, and those of alumina were *E_c_* = 380 GPa and $\rho_{c}$ = 3800 kg/m^3^. The Poisson’s ratio of the plate was assumed to be constant through the thickness, and it equaled 0.3. Equation (2) was used to evaluate the Young’s modulus and density of the plate. The first non-dimensional frequencies $\hat{\omega }$ obtained by the present theory and different methods for some values of the power-law index and length-to-thickness ratios are tabulated in Table 10. It can be seen that a significant agreement between the results of the present theory and different approaches for the first non-dimensional frequencies is found for all length-to-thickness ratios and the power-law index.

**Example** **9.** In this last example, the results of free vibration of a square plate made of Al/Al_2_O_3_ using the proposed theory were compared with those of Brischetto [115] using the exact elasticity solution. The material properties of Al and Al_2_O_3_ were: *E_m_* = 73 GPa, $\nu_{m}$ = 0.3, $\rho_{m}$ = 2800 kg/m^3^, *E_c_* = 380 GPa, $\nu_{c}$ = 0.3, and $\rho_{c}$ = 3800 kg/m^3^. The three cases of dimensions and length-to-thickness ratios were a=b=100, a/h=100; a=b=20, a/h=20, and a=b=5, a/h=5. The mass density and Young’s modulus were obtained by the power-law function. The comparison of the first three non-dimensional frequencies $\tilde{\omega }$ obtained by the proposed theory and those of Brischetto using the exact elasticity solution are given in Table 11. According to Table 11, the results of the proposed theory are in good agreement with those of Brischetto using the exact elasticity solution.

## 6. Conclusions

In this paper, a refined simple first-order shear deformation plate theory was developed for the static bending and free vibration of advanced composite plates such as functionally graded plates. By introducing the distributed shape function to the shear strain, the refined theory accounted for a variable transverse shear strain distribution through the thickness of the plate, and it satisfied the traction free boundary conditions at the top and bottom surfaces of the plate. Moreover, the presented theory retained the simplicity of the FSDT. Analytical solutions were obtained for simply supported FGM plates using the Navier technique. Some numerical examples were carried out to verify the convenience and accuracy of the proposed theory. According to these examples, some remarkable information can be given:

- The proposed theory is efficient and accurate for the static bending and free vibration analysis of FGM plates.
- For FGM plates, the neutral surface is not identical to the mid-plane surface. It moves toward the ceramic surface, and it is different from the isotropic plates.
- The power-law index, aspect ratio, and side-to-thickness ratio have a great effect on the bending behavior and free vibration of FGM plates.

This theory can be applied to the analysis of other structures such as beams and shells made of advanced composite plates. In addition, the proposed theory can be improved by optimizing the distributed shape function to achieve results that are close to the 3D solution, which is a good idea for further work.

## Figures and Tables

**Table 1:** Comparison of center deflections of the isotropic homogeneous plates.

*h*	Classical [1]	3-D [69]	SSDT [52]	Present
0.01	44360.9	44384.7	44383.84	44385.41
0.03	1643.00	1650.94	1650.646	1651.169
0.10	44.3609	46.7443	46.65481	46.81271

**Table 2:** Comparison of distribution of stress across the depth of isotropic homogeneous plates.

*h*	*z*/*h*	$\sigma_{x}$			$\tau_{xy}$		
		3-D [69]	SSDT [52]	Present	3-D [69]	SSDT [52]	Present
0.01	0.5	2873.3	2873.39	2873.51	1949.6	1949.36	1948.61
	0.4	2298.6	2298.57	2298.86	1559.2	1559.04	1558.85
	0.3	1723.9	1723.84	1724.22	1169.1	1168.99	1169.09
	0.2	1149.2	1149.18	1149.58	779.3	779.18	779.33
	0.1	574.6	574.58	574.93	389.6	389.55	389.56
	0.0	0.0	0.00	0.00	0.0	0.00	0.00
0.03	0.5	319.40	319.445	319.279	217.11	217.156	216.512
	0.4	255.41	255.415	255.429	173.26	173.282	173.205
	0.3	191.49	191.472	191.580	129.75	129.682	129.897
	0.2	127.63	127.603	127.731	86.41	86.313	86.592
	0.1	63.80	63.788	63.881	43.18	43.112	43.285
	0.0	0.00	0.00	0.00	0.00	0.000	0.000
0.1	0.5	28.890	28.9307	28.7351	19.920	20.0476	19.4861
	0.4	22.998	23.0055	22.9887	15.606	15.6459	15.5885
	0.3	17.182	17.1660	17.2422	11.558	11.4859	11.6909
	0.2	11.423	11.3994	11.4958	7.642	7.5315	7.7933
	0.1	5.702	5.6858	5.7493	3.803	3.7265	3.8957
	0.0	0.000	0.000	0.000	0.000	0.000	0.000

**Table 3:** Non-dimensional displacements and stress of an FGM square plate under uniform load (*a/h* = 10).

*p*	Source	$\bar{w}$	${\bar{\sigma }}_{x}$	${\bar{\sigma }}_{y}$	${\bar{\tau }}_{yz}$	${\bar{\tau }}_{xz}$	${\bar{\tau }}_{xy}$
Ceramic	SSDT [52]	0.4665	2.8932	1.9103	0.4429	0.5114	1.2850
	Present	0.4681	2.8732	1.9155	0.4665	0.5386	1.2993
1	SSDT [52]	0.9287	4.4745	2.1692	0.5446	0.5114	1.1143
	Present	0.9262	4.4408	2.1768	0.5010	0.4705	1.1221
2	SSDT [52]	1.1940	5.2296	2.0338	0.5734	0.4700	0.9907
	Present	1.1863	5.1853	2.0442	0.4757	0.3899	1.0000
3	SSDT [52]	1.3200	5.6108	1.8593	0.5629	0.4367	1.0047
	Present	1.3081	5.5577	1.8720	0.4452	0.3454	1.0162
4	SSDT [52]	1.3890	5.8915	1.7197	0.5346	0.4204	1.0298
	Present	1.3747	5.8316	1.7338	0.4198	0.3301	1.0430
5	SSDT [52]	1.4356	6.1504	1.6104	0.5031	0.4177	1.0451
	Present	1.4211	6.0858	1.6253	0.4014	0.3333	1.0593
6	SSDT [52]	1.4727	6.4043	1.5214	0.4755	0.4227	1.0536
	Present	1.4593	6.3365	1.5365	0.3901	0.3468	1.0685
7	SSDT [52]	1.5049	6.6547	1.4467	0.4543	0.4310	1.0589
	Present	1.4936	6.5849	1.4615	0.3846	0.3649	1.0743
8	SSDT [52]	1.5343	6.8999	1.3829	0.4392	0.4399	1.0628
	Present	1.5255	6.8288	1.3973	0.3836	0.3842	1.0785
9	SSDT [52]	1.5617	7.1383	1.3283	0.4291	0.4481	1.0662
	Present	1.5556	7.0665	1.3423	0.3858	0.4029	1.0821
10	SSDT [52]	1.5876	7.3689	1.2820	0.4227	0.4552	1.0694
	Present	1.5841	7.2965	1.2954	0.3900	0.4200	1.0855
Metal	SSDT [52]	2.5327	2.8932	1.9103	0.4429	0.5114	1.2850
	Present	2.5413	2.8732	1.9155	0.4665	0.5386	1.2993

**Table 4:** Non-dimensional displacements and stress of an FGM square plate under sinusoidal load (*a/h* = 10).

*p*	Source	$\bar{w}$	${\bar{\sigma }}_{x}$	${\bar{\sigma }}_{y}$	${\bar{\tau }}_{yz}$	${\bar{\tau }}_{xz}$	${\bar{\tau }}_{xy}$
Ceramic	SSDT [52]	0.2960	1.9955	1.3121	0.2132	0.2462	0.7065
	Present	0.2971	1.9758	1.3172	0.2205	0.2546	0.7092
1	SSDT [52]	0.5889	3.0870	1.4894	0.2622	0.2462	0.6110
	Present	0.5872	3.0537	1.4969	0.2369	0.2224	0.6125
2	SSDT [52]	0.7573	3.6094	1.3954	0.2763	0.2265	0.5441
	Present	0.7520	3.5657	1.4057	0.2249	0.1843	0.5459
3	SSDT [52]	0.8377	3.8742	1.2748	0.2715	0.2107	0.5525
	Present	0.8295	3.8218	1.2873	0.2105	0.1633	0.5547
4	SSDT [52]	0.8819	4.0693	1.1783	0.2580	0.2029	0.5667
	Present	0.8721	4.0102	1.1923	0.1984	0.1561	0.5693
5	SSDT [52]	0.9118	4.2488	1.1029	0.2429	0.2017	0.5755
	Present	0.9018	4.1849	1.1176	0.1898	0.1576	0.5783
6	SSDT [52]	0.9356	4.4244	1.0417	0.2296	0.2041	0.5803
	Present	0.9264	4.3574	1.0566	0.1844	0.1639	0.5833
7	SSDT [52]	0.9562	4.5971	0.9903	0.2194	0.2081	0.5834
	Present	0.9485	4.5281	1.0050	0.1818	0.1725	0.5864
8	SSDT [52]	0.9750	4.7661	0.9466	0.2121	0.2124	0.5856
	Present	0.9690	4.6959	0.9609	0.1814	0.1817	0.5887
9	SSDT [52]	0.9925	4.9303	0.9092	0.2072	0.2164	0.5875
	Present	0.9883	4.8593	0.9230	0.1824	0.1905	0.5907
10	SSDT [52]	1.0089	5.0890	0.8775	0.2041	0.2198	0.5894
	Present	1.0065	5.0175	0.8908	0.1844	0.1986	0.5926
Metal	SSDT [52]	1.6070	1.9955	1.3121	0.2132	0.2462	0.7065
	Present	1.6129	1.9758	1.3172	0.2205	0.2546	0.7092

**Table 5:** Non-dimensional deflection and stress of Al/Al_2_O_3_ square plates under sinusoidal loads.

*p*	Source	${\bar{\sigma }}_{x}$			$\bar{w}$		
		*a*/*h* = 4	*a*/*h* = 10	*a*/*h* = 100	*a*/*h* = 4	*a*/*h* = 10	*a*/*h* = 100
1	Quasi-3D [104]	0.5910	1.4917	14.9440	0.7020	0.5868	0.5648
	Quasi-3D [105]	0.5911	1.4917	14.9450	0.7020	0.5868	0.5647
	Quasi-3D [106]	0.5925	1.4945	14.9690	0.6997	0.5845	0.5624
	Quasi-3D [107]	0.6221	1.5064	14.9690	0.7171	0.5875	0.5625
	Quasi-3D [108]	0.6221	1.5064	14.9690	0.7171	0.5875	0.5625
	S-FSDT [29]	0.5987	1.4968	14.9683	0.7291	0.5890	0.5625
	Present	0.5987	1.4969	14.9687	0.7177	0.5872	0.5625
4	Quasi-3D [104]	0.4340	1.1593	11.7380	1.1095	0.8698	0.8241
	Quasi-3D [105]	0.4330	1.1588	11.7370	1.1108	0.8700	0.8240
	Quasi-3D [106]	0.4404	1.1783	11.9320	1.1178	0.8750	0.8286
	Quasi-3D [107]	0.4877	1.1971	11.9230	1.1585	0.8821	0.8286
	Quasi-3D [108]	0.4877	1.1971	11.9230	1.1585	0.8821	0.8286
	S-FSDT [29]	0.4769	1.1922	11.9222	1.1125	0.8736	0.8286
	Present	0.4769	1.1923	11.9228	1.1027	0.8721	0.8286
10	Quasi-3D [104]	0.3108	0.8467	8.6013	1.3327	0.9886	0.9228
	Quasi-3D [105]	0.3097	0.8462	8.6010	1.3334	0.9888	0.9227
	Quasi-3D [106]	0.3227	1.1783	11.9320	1.3490	0.8750	0.8286
	Quasi-3D [107]	0.3695	0.8965	8.6077	1.3745	1.0072	0.9361
	Quasi-3D [108]	0.3695	0.8965	8.6077	1.3745	1.0072	0.9361
	S-FSDT [29]	0.3563	0.8907	8.9072	1.3178	0.9966	0.9361
	Present	0.3563	0.8908	8.9077	1.3796	1.0065	0.9362

**Table 6:** Non-dimensional deflection of exponential function (E)-FGM rectangular plates.

*a*/*h*	*b*/*a*	Method	*p*					
			0.1	0.3	0.5	0.7	1	1.5
2	1	3D [94]	0.5769	0.5247	0.4766	0.4324	0.3727	0.2890
		Quasi-3D [94]	0.5731	0.5181	0.4679	0.4222	0.3612	0.2771
		Quasi-3D [88]	0.5776	0.5222	0.4716	0.4255	0.3640	0.2792
		HSDT [85]	0.6363	0.5752	0.5195	0.4687	0.4018	0.3079
		S-HSDT [56]	0.6362	0.5751	0.5194	0.4687	0.4011	0.3079
		Present	0.6692	0.6062	0.5460	0.4879	0.4003	0.2786
	2	3D [94]	1.1944	1.0859	0.9864	0.8952	0.7727	0.6017
		Quasi-3D [94]	1.1880	1.0740	0.9701	0.8755	0.7494	0.5758
		Quasi-3D [88]	1.1938	1.0790	0.9748	0.8797	0.7530	0.5785
		HSDT [85]	1.2776	1.1553	1.0441	0.9431	0.8093	0.6238
		S-HSDT [56]	1.2775	1.1553	1.0441	0.9431	0.8086	0.6238
		Present	1.3239	1.1928	1.0674	0.9454	0.7578	0.5958
	3	3D [94]	1.4430	1.3116	1.1913	1.0812	0.9334	0.7275
		Quasi-3D [94]	1.4354	1.2977	1.1722	1.0580	0.9057	0.6962
		Quasi-3D [88]	1.4419	1.3035	1.1774	1.0626	0.9096	0.6991
		HSDT [85]	1.5341	1.3874	1.2540	1.1329	0.9725	0.7506
		S-HSDT [56]	1.5340	1.3873	1.2540	1.1329	0.9719	0.7506
		Present	1.5843	1.4255	1.2734	1.1253	0.8965	0.6766
4	1	3D [94]	0.3490	0.3168	0.2875	0.2608	0.2253	0.1805
		Quasi-3D [94]	0.3475	0.3142	0.2839	0.2563	0.2196	0.1692
		Quasi-3D [88]	0.3486	0.3152	0.2848	0.2571	0.2203	0.1697
		HSDT [85]	0.3602	0.3259	0.2949	0.2668	0.2295	0.1785
		S-HSDT [56]	0.3602	0.3259	0.2949	0.2668	0.2295	0.1785
		Present	0.3651	0.3257	0.2879	0.2507	0.1917	0.1088
	2	3D [94]	0.8153	0.7395	0.6708	0.6085	0.5257	0.4120
		Quasi-3D [94]	0.8120	0.7343	0.6635	0.5992	0.5136	0.3962
		Quasi-3D [88]	0.8145	0.7365	0.6655	0.6009	0.5151	0.3973
		HSDT [85]	0.8325	0.7534	0.6819	0.6173	0.5319	0.4150
		S-HSDT [56]	0.8325	0.7534	0.6819	0.6173	0.5319	0.4150
		Present	0.8374	0.7440	0.6543	0.5659	0.5239	0.4240
	3	3D [94]	1.0134	0.9190	0.8335	0.7561	0.6533	0.5121
		Quasi-3D [94]	1.0094	0.9127	0.8248	0.7449	0.6385	0.4927
		Quasi-3D [88]	1.0124	0.9155	0.8272	0.7470	0.6404	0.4941
		HSDT [85]	1.0325	0.9345	0.8459	0.7659	0.6601	0.5154
		S-HSDT [56]	1.0325	0.9345	0.8459	0.7659	0.6601	0.5154
		Present	1.0370	0.9205	0.8088	0.7985	0.6209	0.5708

**Table 7:** The first six non-dimensional frequencies $\omega^{*}$ for square isotropic homogeneous plates.

*a*/*h*	Source	Mode					
		1	2	3	4	5	6
1000	PS-6 [27]	2.000	5.000	5.000	8.000	10.000	10.000
	PS-8a [27]	2.000	5.000	5.000	8.000	10.000	10.000
	PS-8b [27]	2.000	5.000	5.000	8.000	10.000	10.000
	Leissa [2]	2.000	5.000	5.000	8.000	10.000	10.000
	Liew et al. [12]	2.000	5.000	5.000	8.000	10.000	10.000
	Present	2.096	5.241	5.241	8.386	10.482	10.482
5	PS-12 [27]	1.768	3.868	3.868	5.596	6.615	6.615
	PS-14a [27]	1.768	3.868	3.868	5.594	6.611	6.611
	PS-14b [27]	1.807	4.000	4.000	5.807	6.867	6.867
	Liew et al. [12]	1.768	3.866	3.866	5.588	6.601	6.601
	Raju [11]	1.768	3.876	3.876	5.600	6.683	-
	Present	1.843	4.010	4.010	5.779	6.817	6.817

**Table 8:** The first two non-dimensional frequencies $\hat{\omega }$ of isotropic Al/Al_2_O_3_ square plates.

Mode	*a*/*h*	Method	*p*				
			0	0.5	1	4	10
1	2	Quasi-3D [79]	0.9400	0.8233	0.7477	0.5997	0.5460
		S-FSDT [29]	0.9265	0.8062	0.7333	0.6116	0.5644
		Present	0.9114	0.8099	0.7445	0.6165	0.5417
	5	Quasi-3D [79]	0.2121	0.1819	0.1640	0.1383	0.1306
		S-FSDT [29]	0.2112	0.1805	0.1631	0.1397	0.1324
		Present	0.2100	0.1808	0.1639	0.1401	0.1304
	10	Quasi-3D [79]	0.0578	0.0492	0.0443	0.0381	0.0364
		S-FSDT [29]	0.0577	0.0490	0.0442	0.0382	0.0366
		Present	0.0576	0.0490	0.0443	0.0383	0.0364
2	2	Quasi-3D [79]	1.7406	1.5425	1.4078	1.1040	0.9847
		S-FSDT [29]	1.7045	1.4991	1.3706	1.1285	1.0254
		Present	1.6667	1.5088	1.4001	1.1411	0.9710
	5	Quasi-3D [79]	0.4658	0.4040	0.3644	0.3000	0.2790
		S-FSDT [29]	0.4618	0.3978	0.3604	0.3049	0.2856
		Present	0.4570	0.3989	0.3637	0.3064	0.2780
	10	Quasi-3D [79]	0.1381	0.1180	0.1063	0.0905	0.0859
		S-FSDT [29]	0.1376	0.1173	0.1059	0.0911	0.0867
		Present	0.1371	0.1174	0.1062	0.0913	0.0858

**Table 9:** Comparison of the first four non-dimensional frequencies $\bar{\omega }$ of rectangular plate (b/a=2).

*a*/*h*	Mode (*m*, *n*)	Method	*p*						
			0	0.5	1	2	5	8	10
5	1 (1,1)	FSDT [24]	3.4409	2.9322	2.6473	2.4017	2.2528	2.1985	2.1677
		TSDT [36]	3.4412	2.9347	2.6475	2.3949	2.2272	2.1697	2.1407
		SSDT [58]	3.4416	2.9350	2.6478	2.3948	2.2260	2.1688	2.1403
		Present	3.4277	2.9351	2.6562	2.4127	2.2517	2.1823	2.1450
	2 (1,2)	FSDT [24]	5.2802	4.5122	4.0773	3.6953	3.4492	3.3587	3.3094
		TSDT [36]	5.2813	4.518	4.0781	3.6805	3.3938	3.2964	3.2514
		SSDT [58]	5.2822	4.5187	4.0787	3.6804	3.3914	3.2947	3.2506
		Present	5.2507	4.5188	4.0974	3.7202	3.4469	3.3233	3.2599
	3 (1,3)	FSDT [24]	8.0710	6.9231	6.2636	5.6695	5.2579	5.1045	5.0253
		TSDT [36]	8.0749	6.9366	6.2663	5.6390	5.1425	4.9758	4.9055
		SSDT [58]	8.0772	6.9384	6.2678	5.6391	5.1378	4.9727	4.9044
		Present	8.0073	6.9378	6.3078	5.7239	5.2528	5.0298	4.9212
	4 (2,1)	FSDT [24]	9.7416	8.6926	7.8711	7.1189	6.5749	5.9062	5.7518
		TSDT [36]	10.1164	8.7138	7.8762	7.0751	6.4074	6.1846	6.0954
		SSDT [58]	10.1201	8.7167	7.8787	7.0756	6.4010	6.1806	6.0942
		Present	10.0142	8.7147	7.9376	7.2005	6.5674	6.2611	6.1159
10	1 (1,1)	FSDT [24]	3.6518	3.0983	2.7937	2.5386	2.3998	2.3504	2.3197
		TSDT [36]	3.6518	3.0990	2.7937	2.5364	2.3916	2.3411	2.3110
		SSDT [58]	3.6519	3.0991	2.7937	2.5364	2.3912	2.3408	2.3108
		Present	3.6477	3.0991	2.7962	2.5419	2.3994	2.3452	2.3124
	2 (1,2)	FSDT [24]	5.7693	4.8997	4.4192	4.0142	3.7881	3.7072	3.6580
		TSDT [36]	5.7694	4.9014	4.4192	4.0090	3.7682	3.6846	3.6368
		SSDT [58]	5.7697	4.9016	4.4194	4.0089	3.7673	3.6839	3.6365
		Present	5.7594	4.9017	4.4256	4.0224	3.7872	3.6946	3.6403
	3 (1,3)	FSDT [24]	9.1876	7.8145	7.0512	6.4015	6.0247	5.8887	5.8086
		TSDT [36]	9.1880	7.8189	7.0515	6.3886	5.9765	5.8341	5.7575
		SSDT [58]	9.1887	7.8194	7.0519	6.3885	5.9742	5.8324	5.7566
		Present	9.1632	7.8197	7.0674	6.4217	6.0226	5.8583	5.7658
	4 (2,1)	FSDT [24]	11.8310	10.0740	9.0928	8.2515	7.7505	7.5688	7.4639
		TSDT [36]	11.8315	10.0810	9.0933	8.2309	7.6731	7.4813	7.3821
		SSDT [58]	11.8326	10.0818	9.0940	8.2306	7.6696	7.4787	7.3808
		Present	11.7909	10.0823	9.1193	8.2845	7.7472	7.5199	7.3952
20	1 (1,1)	FSDT [24]	3.7123	3.1456	2.8352	2.5777	2.4425	2.3948	2.3642
		TSDT [36]	3.7123	3.1458	2.8352	2.5771	2.4403	2.3923	2.3619
		SSDT [58]	3.7123	3.1458	2.8353	2.5771	2.4401	2.3922	2.3618
		Present	3.7112	3.1457	2.8358	2.5785	2.4423	2.3933	2.3622
	2 (1,2)	FSDT [24]	5.9198	5.0175	4.5228	4.1115	3.8939	3.8170	3.7681
		TSDT [36]	5.9199	5.0180	4.5228	4.1100	3.8884	3.8107	3.7622
		SSDT [58]	5.9199	5.0180	4.5228	4.1100	3.8881	3.8105	3.7621
		Present	5.9171	5.0179	4.5244	4.1136	3.8936	3.8134	3.7631
	3 (1,3)	FSDT [24]	9.5668	8.1121	7.3132	6.6471	6.2903	6.1639	6.0843
		TSDT [36]	9.5669	8.1133	7.3132	6.6433	6.2760	6.1476	6.0690
		SSDT [58]	9.5671	8.1135	7.3133	6.6432	6.2753	6.1471	6.0688
		Present	9.5598	8.1133	7.3176	6.6527	6.2896	6.1547	6.0714
	4 (2,1)	FSDT [24]	12.4560	10.5660	9.5261	8.6572	8.1875	8.0207	7.9166
		TSDT [36]	12.4562	10.5677	9.5261	8.6509	8.1636	7.9934	7.8909
		SSDT [58]	12.4565	10.5680	9.5263	8.6508	8.1624	7.9925	7.8905
		Present	12.4443	10.5679	9.5336	8.6668	8.1863	8.0054	7.8950

**Table 10:** Comparison of first non-dimensional frequencies $\hat{\omega }$ of an Al/Al_2_O_3_ square plate.

*a*/*h*	Method		*n* = 0	*n* = 0.5	*n* = 1	*n* = 4	*n* = 10
2	Analytical	2D-HOT [34]	0.9400	0.8232	0.7476	0.5997	0.5460
		S-HSDT [34]	0.9297	0.8110	0.7356	0.5924	0.5412
		FSDT-IGA [34]	0.9265	0.8060	0.7330	0.6111	0.5640
	Meshless	S-FSDT [35]	0.9270	0.8070	0.7350	0.6136	0.5652
	Present	RS-FSDT	0.9114	0.8097	0.7442	0.6161	0.5412
10	Analytical	2D-HOT [34]	0.0578	0.0492	0.0443	0.0381	0.0364
		S-HSDT [34]	0.0577	0.0490	0.0442	0.0381	0.0364
		FSDT-IGA [34]	0.0577	0.0490	0.0442	0.0382	0.0366
	Meshless	S-FSDT [35]	0.0575	0.0489	0.0442	0.0383	0.0366
	Present	RS-FSDT	0.0576	0.0490	0.0442	0.0382	0.0364
20	Analytical	2D-HOT [34]	0.0148	0.0125	0.0113	0.0098	0.0094
		S-HSDT [34]	0.0146	0.0124	0.0112	0.0097	0.0093
		FSDT-IGA [34]	0.0148	0.0125	0.0113	0.0098	0.0094
	Meshless	S-FSDT [35]	0.0148	0.0125	0.0111	0.0098	0.0094
	Present	RS-FSDT	0.0148	0.0125	0.0113	0.0098	0.0094

**Table 11:** Comparison of first three non-dimensional frequencies $\tilde{\omega }$ of an Al/Al_2_O_3_ square plate.

*a*/*h*	Mode (*m*, *n*)	Method	*p*			
			0	0.5	1	2
100	(1,1)	Exact solution [115]	5.9713	5.0502	4.5529	4.1453
		Present	5.9710	5.0492	4.5526	4.1451
	(2,2)	Exact solution [115]	23.860	20.182	18.195	16.564
		Present	23.857	20.176	18.191	16.561
	(3,3)	Exact solution [115]	53.592	45.338	40.874	37.206
		Present	53.576	45.316	40.858	37.189
20	(1,1)	Exact solution [115]	5.9219	5.0126	4.5193	4.1118
		Present	5.9171	5.0074	4.5144	4.1064
	(2,2)	Exact solution [115]	23.108	19.603	17.681	16.054
		Present	23.038	19.539	17.610	15.974
	(3,3)	Exact solution [115]	50.055	42.605	38.447	34.813
		Present	49.742	42.325	38.129	34.453
5	(1,1)	Exact solution [115]	5.3036	4.5316	4.0923	3.6943
		Present	5.2507	4.4844	4.0382	3.6331
	(2,2)	Exact solution [115]	16.882	14.644	13.278	11.876
		Present	16.467	14.274	12.847	11.387
	(3,3)	Exact solution [115]	30.318	26.597	24.217	21.574
		Present	29.249	27.544	24.819	21.775
